# 

**Published:** 1858-12

**Authors:** 


### Contents of Hall's Journal of Health.

*Ten Numbers of Vol. V.*, 1858—*From Jan. to October*, *inclusive—Sent, post-paid, for* 80 *cts.*

| | PAGE |
|---|---|
| A Pure Joy | 16 |
| Antidote for Yellow Fever | 48 |
| Aids to Digestion | 63, 232 |
| Air and Exercise | 71, 152 |
| Alum in Bread | 90 |
| ABERNETHY Cured of Dyspepsia | 130 |
| Bible Health-teachings | 5, 97 |
| Be Thankful | 21 |
| Best Student | 27 |
| Blacking Boots | 28 |
| Breakfast, Early | 33 |
| Best Preachers | 36 |
| Bathing | 40 |
| Bread | 42, 90, 214 |
| Blessed Charity | 75 |
| Borden's Condensed Milk | 107 |
| Benton, Thomas H., Death | 121 |
| Bites, Poisonous, Cured | 134 |
| Boils, Cure of | 135, 230 |
| Balm of Gilead | 144 |
| Children Training | 13, 19, 37, 109 |
| Clerical Exposures | 15, 117, 176, 183 |
| Consumption | 23, 185 |
| Constipation Avoided | 183 |
| Condensed Milk | 107 |
| Chat about Health | 115, 182 |
| Cancer | 121 |
| Coffee and Tea | 131, 167, 237 |
| Dyspepsia | 32, 63, 123–5, 130–2 |
| Drunkenness Cured | 156 |
| Danger to Orators | 61, 133, 140 |
| Doctors | 109, 119, 143, 230, 233 |
| Employments, Health of | 26, 191 |
| Eating | 17, 33, 73, 124, 128, 192 |
| Exercise and Air | 71, 124 |
| Edwards, Jonathan, Eating Trials | 128 |
| Epilepsy | 173 |
| Early Rising | 203 |
| Fever and Ague Prevented | 30, 217 |
| Food of Infants | 99 |
| Fruits, Use of | 94, 123, 141, 202 |
| Forethought | 197 |
| Gratuitous Medical Advice | 25, 143 |
| Greatness and Health | 38 |
| Gluttony | 89 |
| Going Down | 117 |
| Health of Employments | 26, 191 |
| Heart Affections | 162 |
| Health a Duty | 39, 49, 115, 235 |
| Hair Specifics | 179 |
| Health of Cities | 72, 97 |
| Horseback Exercise | 113 |
| Hard Study Healthful | 164 |
| How to Get Sick | 138 |
| Insanity | 59 |
| Inhalation, Medicated | 72 |
| Infants' Food | 99 |
| Insect Bites Cured | 134 |
| Inverted Toe-nails | 239 |
| Liquor and Tobacco | 18, 88, 91, 133 |
| Life-Long Lunatic | 24 |
| Late Suppers | 31 |
| Longevity of Slaves | 43 |
| Loss of Voice | 74 |
| Liver and Stomach | 135 |
| Leaning on Providence | 144 |
| Marriage | 11 |
| Miscellanies | 17, 145 |
| Monumental Heights | 28 |
| Malarial Precautions | 30 |
| Ministerial Support | 35 |
| Medicated Inhalation | 72 |
| Milk, Bad | 99, 105 |
| Moths Exterminated | 114 |
| Novel Reading Pernicious | 46, 112 |
| Sight Injured | 194 |
| New York Mortuary | 72 |
| Nurses and Nursing | 87 |
| Nutrition and Stimulation | 91 |
| Night Air | 216 |
| Out Door Safety | 16 |
| Orchard Planting | 52 |
| Oratory, Dangers of | 15, 61, 117, 140 |
| Over-eating | 73 |
| Onions, Nutritious | 99 |
| Our Troubles | 110 |
| One Calling | 143 |
| Pigs and Men | 17, 133 |
| Pulpit Power | 34, 133 |
| Passing Away | 41 |
| Parlor Ornaments | 50 |
| Planting Trees | 52 |
| Pills by the Bushel | 95 |
| Poor Man's Book | 97 |
| Poisonous Milk | 105 |
| Potato, New | 134 |
| Patent Medicine Swindles | 134, 142 |
| Providence Reposed in | 144 |
| Paste | 146 |
| Reading, Fictitious | 46 |
| Riddance of Rats and Vermin | 49, 92, 144 |
| Relapses | 116 |
| Recreations | 121, 187, 211 |
| School Children | 19, 154, 241 |
| Suppers, Late | 31, 139, 169 |
| Safe Reading | 46 |
| Sea Voyages | 64 |
| Sabbath Hygiene | 65, 188 |
| Sneezing and Stupidities | 86, 131, 190 |
| Stimulation and Nutrition | 91 |
| Sleep, Delicious | 108, 163, 238 |
| Sprains | 153 |
| Saleratus and Soda Hurtful | 124 |
| Stitch in the Side | 131 |
| Stings of Insects Cured | 134 |
| Sewing Machines | 135 |
| Stomach Managed | 136 |
| Stammering | 209 |
| Things to Avoid | 14, 33 |
| Tobacco and Liquor | 18, 133 |
| Thanksgiving Dinner | 20 |
| Thankfulness, Cause for | 21 |
| Trouble | 119, 160, 161 |
| Throat-Ail | 77, 132 |
| Temperate Eating | 136,128, 228 |
| Victoria as a Mother | 41 |
| Vinegar, the Best | 63 |
| Voice, Lost | 74 |
| Vermin Riddance | 49, 92, 140, 144 |
| Warm and Cold Baths | 40 |
| Warts Cured | 134 |
| Yellow Fever Antidote | 48 |

